# Preoperative Steroid Use in Rhegmatogenous Retinal Detachment Associated with Choroidal Detachment after 23-Gauge Vitrectomy

**DOI:** 10.1155/2020/6707239

**Published:** 2020-05-02

**Authors:** Jianhua Wu, Rui Zhang, Junwen He, Changzhong Xu, Zhaohui Li

**Affiliations:** Retinal & Vitreous Diseases Department of Wuhan Aier Eye Hospital, Aier Ophthalmic College of Central-South University, Wuhan 430064, China

## Abstract

**Background:**

Rhegmatogenous retinal detachment associated with choroidal detachment (RRDCD) is rare and the prognosis is poor. This retrospective study evaluated the effect of preoperative steroid on the clinical outcome of patients with RRDCD receiving 23-gauge pars plana vitrectomy (PPV).

**Methods:**

Sixty-six patients (67 eyes) with diagnosed RRDCD underwent 23-gauge PPV. The patients assigned to receive systemic or subtenon injection of preoperative steroids were considered Group A (35 eyes) and did not receive are considered Control Group B (32 eyes). Most patients in Group A received subtenon injection of glucocorticoids. The cyclodialysis angle was measured with ultrasound biomicroscopy. Preoperative, intraoperative, and postoperative data were compared.

**Results:**

The rates of retinal reattachment in Group A after the first and second operations were 68.8% (24/35 eyes) and 91.43% (32/35 eyes), respectively, which were not significantly different from that of Group B (78.1%, 25/32 eyes; 96.6%, 31/32 eyes). The logMAR (logarithm of the minimum angle of resolution) visual acuity in Group A (1.63 ± 0.75) was similar to that of Group B (1.34 ± 0.74). Postoperative intraocular pressure and ocular hypertension in Group A (17.94 ± 9.82 mmHg and 37.1%, respectively; 13/35 eyes) were comparable to that of Group B (20.93 ± 10.21 mmHg and 56.3%; 18/32 eyes). Logistic regression analysis showed that postoperative reattachment was negatively associated with preoperative cyclodialysis angle as measured with ultrasound biomicroscopy (*P*=0.048) but was not significantly associated with preoperative steroid use (*P*=0.907).

**Conclusions:**

Preoperative steroid use does not improve retinal reattachment and visual acuity in patients with RRDCD after 23-gauge PPV. Preoperative measurement of the cyclodialysis angle with ultrasound biomicroscopy may be useful for predicting clinical outcomes.

## 1. Introduction

Rhegmatogenous retinal detachment (RRD) associated with choroidal detachment (RRDCD) is an uncommon type of RRD, characterized by low intraocular pressure (IOP), decreased visual acuity, and secondary uveal inflammation [1]. In the clinical setting, retinal detachment as well as choroidal and ciliary detachment can be observed using ophthalmoscopy, ultrasound biomicroscopy, and B-type ultrasonography.

RRDCD is rare, accounting for approximately 2.0 to 8.6% of all RRD cases [2–4]. The risk factors associated with RRDCD are old age, high myopia, multiple retinal breaks, and macular hole [5–7]. Independent risk factors include extent of retinal detachment, high myopia, and low IOP [7]. Despite its rarity, RRDCD progresses rapidly, with a high rate of recurrence, and is commonly associated with poor prognosis [1, 8]. The poor rate of postoperative retinal reattachment in RRDCD is due to the development of proliferative vitreoretinopathy (PVR) [9].

Although the pathogenesis of RRDCD is unclear, it is believed that in RRD, low IOP elicits choroidal exudation that leads to suprachoroidal fluid accumulation, eventually resulting in choroidal and ciliary detachment [4]. In addition, Jarrett [1] hypothesized that ocular inflammation associated with RRD induced the accumulation of exudation in the suprachoroidal space, which caused choroidal and ciliary detachment. Choroidal and ciliary detachment can further disrupt the circulation of the aqueous humor, aggravating RRD by lowering IOP and promoting the development of PVR. We have previously found that low IOP and severe choroidal and retinal detachment are risk factors associated with surgical failure in patients with RRDCD [5]. However, it remains unclear whether ocular inflammation contributes to postoperative outcomes.

It has been reported that the development of PVR is associated with inflammation [10]. Steroids can inhibit the inflammatory reaction and thus may prevent PVR [11]. Steroids may also inhibit the breakdown of the blood-retinal barrier [12] and reduce cell proliferation [13]. Therefore, it is important to investigate the effect of preoperative steroid use on the clinical outcome of patients with RRDCD after vitrectomy.

Sharma et al. [14] reported that preoperative oral administration of steroids improved the rate of retinal reattachment in patients with RRDCD receiving primary vitrectomy. Similarly, Shen et al. [15] found that subtenon injection of triamcinolone acetonide (TA) before vitrectomy improved the clinical outcomes of patients with RRDCD. However, a recent study by Denwattana et al. [16] showed that preoperative steroid treatment did not improve the rate of retinal reattachment or visual acuity at 3 months.

Since steroid use is associated with many side effects such as hypertension, hyperglycemia, osteoporosis, and disorder of lipid metabolism, steroids should be applied cautiously before a beneficial effect in patients with RRDCD is proved. Since 23-gauge pars plana vitrectomy (PPV) has been widely used for the treatment of RRD, it is important to determine whether preoperative steroid use can improve clinical outcomes in patients with RRDCD receiving 23-gauge PPV.

In this retrospective study, we evaluated the effect of preoperative steroid use on the clinical outcome of patients with RRDCD who received 23-gauge PPV.

## 2. Materials and Methods

### 2.1. Patients

The Institutional Review Board of Aier School of Ophthalmology of the Central South University approved this retrospective cohort study. The experiments were conducted in accordance with the Declaration of Helsinki.

The study included 67 eyes of 66 consecutive patients with primary RRDCD, which was treated with 23-gauge minimally invasive vitreous surgery at Wuhan Aier Eye Hospital between September 2015 and October 2017. Patients with any of the following were excluded: secondary RRDCD after vitreous surgery; eye trauma; aged more than 80 years; severe diseases such as hypertension, cardiovascular diseases, diabetes, and renal insufficiency; intolerant of the surgery; and lost to follow-up.

The patients were assigned to two groups as follows. Group A (*n* = 35, eyes of 35 patients) received systemic or subtenon or subconjunctival injection of glucocorticoids, 3 days before surgery. However, Group B (*n* = 32, eyes of 31 patients) were not given glucocorticoid treatment. The choice of preoperative glucocorticoid administration was decided by the general condition of the patient and the tolerance to the side effects of systemic administration of corticosteroids.

The preoperative glucocorticoid regimen included the following: subtenon injection of dexamethasone sodium phosphate (DEX) (5 mg) or triamcinolone acetonide (TA) (40 mg); subconjunctival injection of DEX (2.5 mg); oral administration of prednisone (0.5 mg/kg/d, 3 d); and intravenous injection of methylprednisolone (500 mg/day, 3 d). All the patients received topical administration of tobramycin dexamethasone eye drops (4 times daily; Alcon, USA) and tobramycin dexamethasone eye ointment (once per night; Alcon, USA) until surgery.

### 2.2. Surgery

All patients underwent PPV using a 23-gauge vitrectomy system. Phacoemulsification was performed in patients with severe lens opacity that could affect the surgery. The vitreous was stained with TA during the surgery and was removed as much as possible. For patients with moderate-to-severe choroidal detachment, suprachoroidal puncture was performed at the uppermost part of the detachment, and the suprachoroidal fluid was drained to avoid insufficient filling of the silicone oil. For patients with severe choroidal detachment and low IOP, BSS (balanced salt solution) was injected from the pars plana using a 30 G syringe needle to increase IOP. Then, a transconjunctival puncture was performed at the quadrant with the severe choroid detachment. A long infusion cannula (6 mm) was inserted at the uppermost part of the detachment to release the suprachoroidal fluid and reduce choroid detachment.

### 2.3. Data Collection

The patients' medical histories were collected and compared between the two groups, such as age, gender, and disease onset time. Comprehensive preoperative ocular examinations were recorded, including IOP; best-corrected visual acuity (BCVA); presence of high myopia; presence of macular hole; measurement of cyclodialysis angle with ultrasound biomicroscopy (UBM); extent of the detachment of the choroid, ciliary body, and retina; number and location of retinal breaks; and PVR grade. The cyclodialysis angle was measured with UBM. The apex of the angle was the beginning detachment site of the ciliary body, and the two sides were the inner wall of the sclera and the tangent line of the lateral border of the detached ciliary body. The PVR grade was defined as previously reported by Machemer et al. [17]. The intraoperative information was collected, including surgical procedure, laser retinopexy or retinal cryopexy, intraocular tamponade, and intraoperative complications. The postoperative examinations included IOP, BCVA, postoperative complications, and retinal reattachment.

### 2.4. Statistical Analysis

Analyses were performed using SPSS 13.0 software. Student's *t*-test was used to compare differences in age, BCVA, IOP, and disease onset time between the two groups. A paired *t-*test was used to compare differences in BCVA and IOP within the group. Categorical data were compared using the chi-squared test, including gender, macular hole, high myopia, and recurrence after primary surgery. The Mann–Whitney rank-sum test was used to compare the extent of choroidal detachment, PVR grade, and the extent of retinal detachment. Two-dimensional logistic regression analysis was applied to assess the association of postoperative retinal reattachment with preoperative glucocorticoid use and cyclodialysis angle. Probability values <0.05 were considered statistically significant.

## 3. Results

Sixty-six patients (67 eyes) with RRDCD were included in this study (Table 1). The average age of these patients was 52.03 ± 14.15 years (range, 13–76 y). Thirty-six (54.55%) patients were male and 30 (45.45%) were female. Of the 66 patients enrolled in this study, 35 and 32 were included in groups A and B, respectively. The two groups were statistically similar with regard to the following: age, gender, presence of macular hole, preoperative IOP, the number of retinal breaks, high myopia, extent of retinal detachment and choroidal detachment, cyclodialysis angle, PVR grade, lens status (pseudophakic or aphakic), and disease onset time.

Of the 35 patients in Group A, 23, 5, and 2 received, respectively, subtenon injection of dexamethasone, subconjunctival injection of dexamethasone, and subtenon injection of TA (Table 2). Two and 3 patients also received oral prednisone and intravenous injection of methylprednisolone. All patients in both groups received topical administration of tobramycin dexamethasone eye drops and eye ointment.

All patients in groups A and B underwent 23-gauge pars PPV (Table 3). Of the 35 eyes in Group A, silicon oil tamponade was used in 32 (91.4%) and perfluoropropane (C3F8) tamponade in 3 (8.6%). Of the 32 eyes in Group B, silicon oil tamponade was used in 27 (84.4%) and C3F8 tamponade in 5 (5.6%). In addition, laser retinopexy and retinal cryopexy were performed in 32 (91.4%) and 12 (34.3%) eyes in Group A, respectively, and in 29 (90.6%) and 12 (37.5%) in Group B. Phacoemulsification was performed in 9 (25.71%) eyes in Group A and 7 (21.88%) in Group B. There was no significant difference in the surgical procedures of the two groups.

In Group A, the retinal reattachment rates after the first and second operations were 68.8% (24/35 eyes) and 91.43% (32/35 eyes), respectively (Table 4). In Group B, these rates were 78.1% (25/32 eyes) and 96.6% (31/32 eyes). There was no significant difference in retinal reattachment rates between groups A and B. The recurrent rates of retinal detachment were 5.56%, 22.22%, and 72.22% for mild, moderate, and severe choroidal detachment, respectively. The postoperative BCVA (as measured by the logMAR (logarithm of the minimum angle of resolution)) of Group A (1.63 ± 0.75) and Group B (1.34 ± 0.74) was not significantly different (*P*=0.11). In addition, the postoperative IOP was 17.94 ± 9.82 mmHg in Group A, which was not significantly different from that of Group B (20.93 ± 10.21 mmHg; *P*=0.294). The incidence of ocular hypertension was 37.1% (13/35 eyes) in Group A and 56.3% (18/32 eyes) in Group B (*P*=0.117).

Two-dimensional logistic regression analysis was used to evaluate the association between postoperative retinal reattachment and preoperative glucocorticoid use and cyclodialysis angle (Tables 5 and 6). Overall, the postoperative reattachment was negatively associated with the preoperative cyclodialysis angle (*P*=0.048). No significant association was found between postoperative reattachment and the following: preoperative glucocorticoid use (*P*=0.907); postoperative IOP (*P*=0.076); old age (*P*=0.426); high myopia (*P*=0.197); multiple retinal breaks (*P*=0.644); and PVR grade (*P*=0.118).

## 4. Discussion

In this study, we investigated the effect of preoperative steroid use on the clinical outcomes of patients with RRDCD after 23-gauge PPV. We found that preoperative steroid use did not improve retinal reattachment and visual acuity in these patients. However, postoperative retinal reattachment was negatively associated with preoperative cyclodialysis angle, a parameter that was measured with ultrasound biomicroscopy and reflects disease severity. Our findings suggest that preoperative steroid use may not improve the clinical outcome of patients with RRDCD after 23-gauge PPV, and the preoperative cyclodialysis angle may be used as a prognostic factor. We did not find any significant association between retinal reattachment and old age, high myopia, multiple retinal breaks, or PVR grade, although these factors are associated with RRDCD [5–7].

The clinical outcomes for preoperative steroid use in patients with RRDCD after vitrectomy have been unclear. This is partly because the use of steroids among different studies is inconsistent regarding the types, dosage, administration route, and duration. Sharma et al. [14] conducted a prospective study of patients with RRDCD after 20-gauge PPV, who were also treated with scleral buckling or encircling. The rate of retinal reattachment when preoperative oral prednisolone was applied (81.8%; 1 mg/kg/day for 1 week) tended to be higher compared to the control group not using steroid (66.7%), although the difference was not significant. In addition, Denwattana et al. [16] found that preoperative oral prednisolone (0.5–1 mg/kg/day, 7 d) or subtenon injection of TA (20 or 40 mg) did not improve retinal reattachment rate in patients with RRDCD after PPV. This may be because short-term preoperative steroid use may not be sufficient to inhibit PVR formation, a continuous process that is associated with poor postoperative retinal reattachment [9]. In addition, in Denwattana's study, most patients underwent 23- or 25-gauge PPV, whereas in Sharma's study, most patients received 20-gauge PPV. Whether the surgical procedure affects the clinical outcome remains to be determined.

Shen et al. [15] reported that subtenon TA (40 mg, 5 d) with silicone oil tamponade increased TA concentrations in the vitreous and improved the clinical outcomes of patients with RRDCD after vitrectomy. Wei et al. [18] found that the effect of intraocular/intravitreal injection of steroids was comparable to oral administration of steroids for the treatment of RRDCD. However, in these studies, the PVR grade of the involved eyes was not indicated.

In the present study, we found that preoperative steroid use did not improve the retinal reattachment rate or visual acuity of patients with RRDCD receiving 23-gauge PPV. This is similar to the report of Denwattana et al. [16]. However, the PVR of the eyes of that study (<30% grade C) was not as severe as that of the present population (>50% grade C). The higher PVR grade reflects a severe stage of RRDCD disease and may be associated with poor response to steroid treatment.

Inflammatory mediators and proteins in suprachoroidal fluid may promote PVR formation and affect the outcome of silicone oil tamponade. Therefore, for patients in the present study with moderate-to-severe choroidal detachment, we performed suprachoroidal puncture at the uppermost part of the detachment to drain the choroidal fluid as much as possible. This procedure can reduce inflammatory mediators in the eyes, thus preventing PVR formation. It can also effectively control the volume of silicone oil.

In the present study, we used ultrasound biomicroscopy to measure the cyclodialysis angle, which was used to grade the severity of choroidal detachment as mild, moderate, or severe. We found that the rate of recurrence was higher in patients with more severe choroidal detachment (i.e., 5.56%, 22.22%, and 72.22% for mild, moderate, and severe choroidal detachment, respectively). This suggests that severe choroidal detachment is associated with a higher recurrence rate in patients with RRDCD after primary vitrectomy. In addition, the logistic regression analysis showed that the preoperative cyclodialysis angle was negatively associated with postoperative reattachment. These findings suggest that measuring the cyclodialysis angle with ultrasound biomicroscopy is useful for grading the severity of choroidal detachment and can be used for predicting clinical outcomes in these patients.

We previously found that preoperative IOP is positively associated with the rate of surgical success in patients with RRDCD after vitrectomy [5]. However, it remains unclear whether postoperative IOP affects the clinical outcome of patients with RRDCD receiving vitrectomy. Since postoperative IOP is closely associated with choroidal reattachment, functional recovery of the ciliary body, and the effect of silicone oil tamponade, it is expected that postoperative IOP may affect the clinical outcome of patients after vitrectomy. In the present study, patients who received the preoperative steroid had a lower rate of postoperative ocular hypertension (37.1%) compared to the control group (56.3%), although this was not a statistically significant difference. A study with a larger sample size would have more power to confirm these results. The logistic regression analysis also did not indicate a significant association between postoperative IOP and retinal reattachment.

This study has some limitations, due to its retrospective nature. In addition, although all surgeries were performed by the same experienced surgeon, the type and administration route as well as the duration of preoperative steroids were not consistent among the patients. Therefore, future studies are required to investigate whether the type, administration route, and duration of preoperative steroids may affect the clinical outcomes in patients after 23-gauge PPV. Finally, the follow-up period was relatively short (6 months). It is important to determine whether preoperative steroid use has a long-term effect in patients with RRDCD after 23-gauge PPV.

In summary, we investigated the effect of preoperative steroid use on the clinical outcome of patients with RRDCD after 23-gauge PPV and found that preoperative steroid use did not significantly improve retinal reattachment and visual acuity in these patients. This suggests that a preoperative steroid may not be necessary for these patients. However, we found that the preoperative cyclodialysis angle, determined by ultrasound biomicroscopy, was negatively associated with postoperative retinal reattachment. Therefore, measurement of the cyclodialysis angle using ultrasound biomicroscopy may be useful for predicting clinical outcomes in patients with RRDCD after vitrectomy.

## Figures and Tables

**Table 1:** Demographic and clinical data of patients with RRDCD^*∗*^.

		Group A	Group B	*P* value
Subjects, *n*		35	31	
Age, y		54.83 ± 11.98	48.97 ± 15.81	0.116

Gender	Male	19 (54.29%)	18 (56.25%)	0.872
	Female	16 (45.71%)	14 (43.75%)	

Macular hole		6	1	0.061
Preop IOP, mmHg		5.97 ± 2.89	6.54 ± 3.37	0.47
Retinal holes, *n*		3.17 ± 2.37	2.59 ± 1.78	0.267
High myopia		15 (42.9%)	10 (31.3%)	0.326

RD quadrants	1	1	0	0.098
	2	2	8	
	3	2	2	
	4	30	22	

Choroidal detachment quadrants	1	0	1	0.578
	2	0	0	
	3	3	3	
	4	32	28	

Cyclodialysis angle	Mild	3	6	0.066
	Moderate	8	11	
	Severe	24	15	

PVR grade	A	3 (8.57%)	2 (6.25%)	0.832
	B	14 (40%)	13 (40.63%)	
	C	18 (51.43%)	17 (53.12%)	

Duration of symptoms, d		23.47 ± 15.79	25.22 ± 18.76	0.684
Pseudophakia or aphakia		2	3	0.917

^*∗*^Reported as *n* or *n* (%), unless indicated otherwise.

**Table 2:** Preoperative glucocorticoid use in patients with RRDCD, *n*.

	Group A	Group B
Subjects, *n*	35	32

Subtenon injection DEX^a^	23	0
Subtenon injection TA^b^	2	0

Subconjunctival injection DEX^c^	5	0
Oral prednisone^d^	2	0

IV drop methylprednisolone^e^	3	0
Steroidal eye drop	35	32

^a^5 mg; ^b^40 mg; ^c^2.5 mg; ^d^0.5 mg/kg/d; ^e^500 mg/d for 3 d.

**Table 3:** Surgical procedures in patients with RRDCD^*∗*^.

		Group A	Group B	*P* value
Subjects, *n*		35	32	
23G pars plana vitrectomy		35 (100)	32 (100)	—

Laser retinopexy		32 (91.4)	29 (90.6)	0.754
Retinal cryopexy		12 (34.3)	12 (37.5)	0.784

Vitreous substitute	Silicon oil	32 (91.4)	27 (84.4)	0.608
	C3F8	3 (8.6)	5 (5.6)	

Phacoemulsification		9 (25.71)	7 (21.88)	0.713

^*∗*^Reported as *n* (%), unless indicated otherwise.

**Table 4:** Anatomical and functional outcomes after 23-gauge PPV in patients with RRDCD^*∗*^.

		Group A	Group B	*P* value
Subjects, *n*		35	32	—
Post-op logMAR VA at 3 months		1.63 ± 0.75	1.34 ± 0.74	0.11

Reattachment	After first operation	24 (68.6)	25 (78.1)	0.378
	After second operation	32 (91.43)	31 (96.9)	0.412

Post-op ocular hypertension		13 (37.1)	18 (56.3)	0.117
Post-op IOP, mmHg		17.94 ± 9.82	20.53 ± 10.21	0.294

^*∗*^Reported as *n* (%), unless indicated otherwise. VA, visual acuity.

**Table 5:** Retina reattachment ratios compared with influencing factors of variable description.

	Assignment description
Cyclodialysis angle (X2)	1, cyclodialysis angle <12
	2, 12 < cyclodialysis angle < 22
	3, 22 <cyclodialysis angle

Retina reattachment (Y)	0, surgery failure
	1, surgery success

**Table 6:** Logistic regression analysis.

	B	SE	Wald	DF	Sig.	Exp (B)
Cyclodialysis angle	−0.969	0.491	3.896	1	0.048	0.379
Constant	3.407	1.340	6.468	1	0.011	30.178

## Data Availability

The datasets generated and analyzed during the present study are available from the corresponding author upon reasonable request.
